# Highly-ordered silicon inverted nanocone arrays with broadband light antireflectance

**DOI:** 10.1186/s11671-014-0718-x

**Published:** 2015-01-22

**Authors:** Dong Zhang, Weina Ren, Zhichao Zhu, Haifeng Zhang, Bo Liu, Wangzhou Shi, Xiaomei Qin, Chuanwei Cheng

**Affiliations:** Department of Physics, Shanghai Normal University, No.100 Guilin Road, Shanghai, 200234 PR China; Shanghai Key Laboratory of Special Artificial Microstructure Materials and Technology and School of Physics Science and Engineering, Tongji University, 1239 Siping Road, Shanghai, 200092 PR China

**Keywords:** Inverted nanocone arrays, Antireflection, Nanosphere lithography, Si

## Abstract

In this work, highly-ordered silicon inverted nanocone arrays are fabricated by integration of nanosphere lithography with reactive ion etching (RIE) method. The optical characteristics of as-prepared Si inverted nanocone arrays are investigated both by experiments and simulations. It is found that the Si nanocone arrays present excellent broadband light antireflectance properties, which are attributed to the gradient in the effective refractive index of nanocones and enhanced light trapping owing to optical diffraction. The inverted Si nanocone arrays might find a variety of applications in solar cells and photodetectors.

**Electronic supplementary material:**

The online version of this article (doi:10.1186/s11671-014-0718-x) contains supplementary material, which is available to authorized users.

## Background

Photovoltaic is a promising technology for generating electrical power from the sun on a large scale. The silicon solar cell is presently dominating the solar cell market, owing to the abundance of raw materials, near ideal band gap, and mature fabrication process [1]. However, the major issue with the planar Si is the high light reflectance loss on the interface between the air and Si. Due to the high refractive index (*n* = 3.4) of Si, more than 30% of incident sunlight is scattered or reflected from the Si surface, which has limited the efficient utilization of sunlight. One of the traditional methods to reduce the reflection loss on the surface and enhance the light absorption is to use an anti-reflection (AR) layer [2,3], such as Si_3_N_4_, SiO_2_, etc. However, such AR layer works best only for light with individual wavelength and special incident angle. Texturization of the surface is another efficient way to realize light trapping and absorption enhancement [4-7]. In the past few years, various nanostructures including nanowires [8-10], nanopillars [11], nonopyramids [12,13], and nanocones [14,15] were explored for nanostructured thin film photovoltaic devices with excellent light trapping abilities. All the nanostructures mentioned above can be categorized as ‘positive’ structures with respect to the substrates, that is, the structures protrude out from the substrates into free space. In contrast to the deep research on the ‘positive’ structures, the development of ‘negative’ nanostructure, for instance, nanoholes, is still far behind due to the limited fabrication methods.

In this letter, we report the fabrication of highly-ordered Si inverted nanocone arrays with desired diameters and pitches by a combination of colloid lithography and reactive ion etching route. The photon-trapping process in the nanocone arrays were studied by experimentally and theoretically investigating their optical absorption properties. It was found that strong diffraction of light can enhance the photon-harvesting ability, especially when the diameters of the holes are matched with the optical wavelength.

## Methods

### Si inverted nanocone arrays fabrication

First, the planar Si (MTI, China) wafer was ultrasonicated in deionized (DI) water, acetone, and methanol for 5 min. Then, the substrate was heated in boiling piranha solution (H_2_SO_4_/H_2_O_2_ with a volume ratio of 4:1) for 10 min to remove organic residues. After each cleaning step, the wafer was washed with DI water. After the standard RAC process mentioned above, the surface of the substrate is hydrophobic. A monolayer of closed-packed polystyrene (PS) spheres was fabricated on the Si surface via a self-assembly route. For this experiment, PS beads with diameter of 500 and 1,000 nm have been used. A drop of the colloidal dispersion was put onto the P-type Si wafers, and the water was allowed to evaporate slowly under ambient conditions. The sphere diameter was subsequently reduced slightly via reactive ion etching (RIE) in oxygen (O_2_) plasma. The gas flow was 40 sccm, the power was 40 W, the chamber pressure was 9.8 Pa, and the etching time was 300 s. After that, a 50-nm nickel (Ni) layer was deposited onto the samples by magnetron sputtering method. The power was 70 W and sputtering time was 3,600 s. After the deposition, the PS spheres were dissolved in toluene for 5 ~ 6 h. Then, the samples were washed with DI water. Afterward, the samples were etched by RIE using the mixture of SF_6_ and O_2_. The gas flow of SF_6_ and O_2_ was 70 and 10 sccm, respectively, the power was 150 W, and the chamber pressure was 9.8 Pa. Since the selective properties of RIE with different gas, the most removed part is only Si but not Ni. The depth of the nanohole could be adjusted by well controlling the etching time. Finally, the remained Ni layer was removed by soaking the sample in HCl solution (HCl:DI = 1:3) for 15 min. Then, the samples were rinsed with DI water and dried with nitrogen.

### Characterization

The morphologies of the Si inverted nanocone arrays were characterized by scanning electron microscope (SEM, Hitachi S-4800, Hitachi, Tokyo, Japan). The hemispherical reflectance of the samples was measured by an UV-vis spectrometer (Zolix Instruments Co., Ltd, Beijing, China). The numerical simulations for the reflectance spectra and the spatial distributions of electric field intensity were performed based on a rigorous coupled wave analysis (RCWA) method and three-dimensional finite-difference-time-domain (FDTD) method.

## Results and discussion

**Figure 1:**
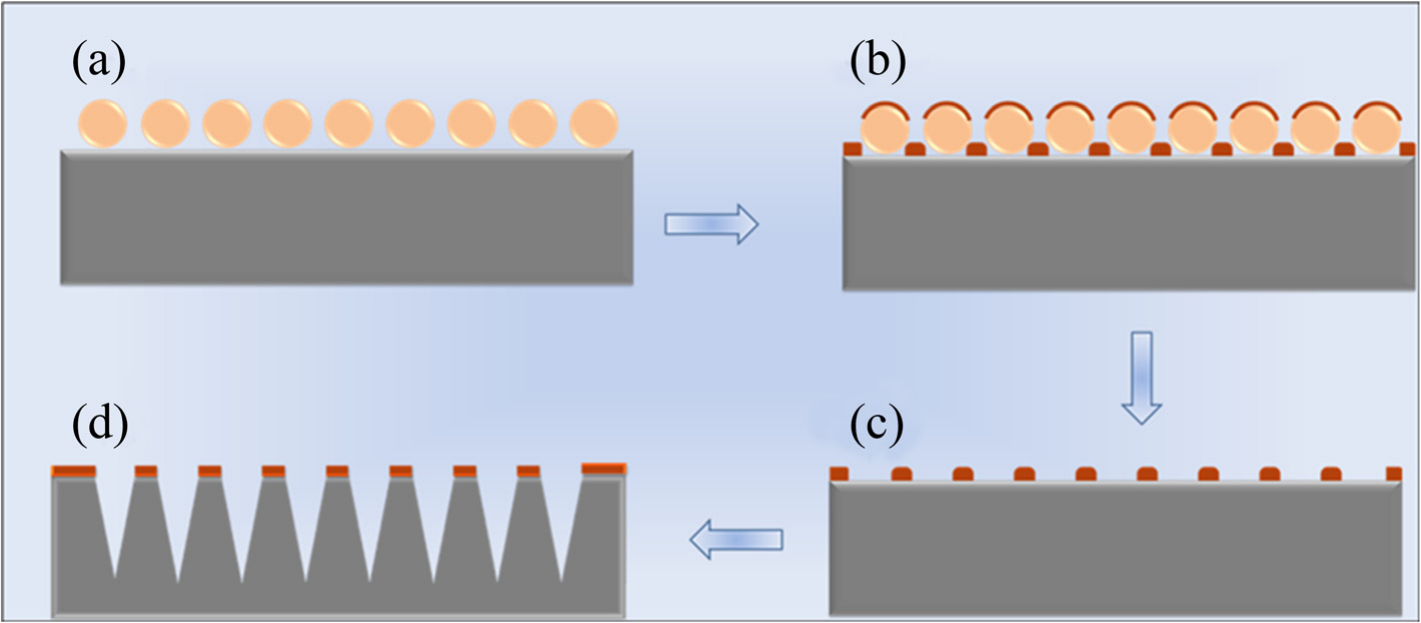
**Scheme of the fabrication procedures of Si inverted nanocone arrays. (a)** Fabrication of PS spheres monolayer, **(b)** Ni film deposition, **(c)** PS spheres removal, and **(d)** RIE etching.

The fabrication procedures of the periodical Si inverted nanocone arrays are illustrated in Figure 1. First, a monolayer of closed-packed polystyrene spheres is fabricated on the hydrophilic P-type Si surface which was cleaned by a standard RAC process. The size of PS sphere was reduced by RIE with O_2_, forming a 2D non-close-packed PS template, which was used as a nanopatterned mask, as shown in Figure 1a. Second, 50 nm of Ni film was deposited on the PS spheres and the interstitials. By removing the PS sphere template, a periodical Ni nanohole arrays was obtained, as shown in Figure 1c. The Ni film with periodical nanohole arrays was used as a hard mask for RIE in the SF_6_/O_2_ plasma. The Si nanohole's depth can be controlled by adjusting the etching time, while the diameters and pitches of the nanoholes were defined by the PS nanosphere patterns, as shown in Figure 1d. The remained Ni mask layer can be removed by soaking the samples in HCl solution.

**Figure 2:**
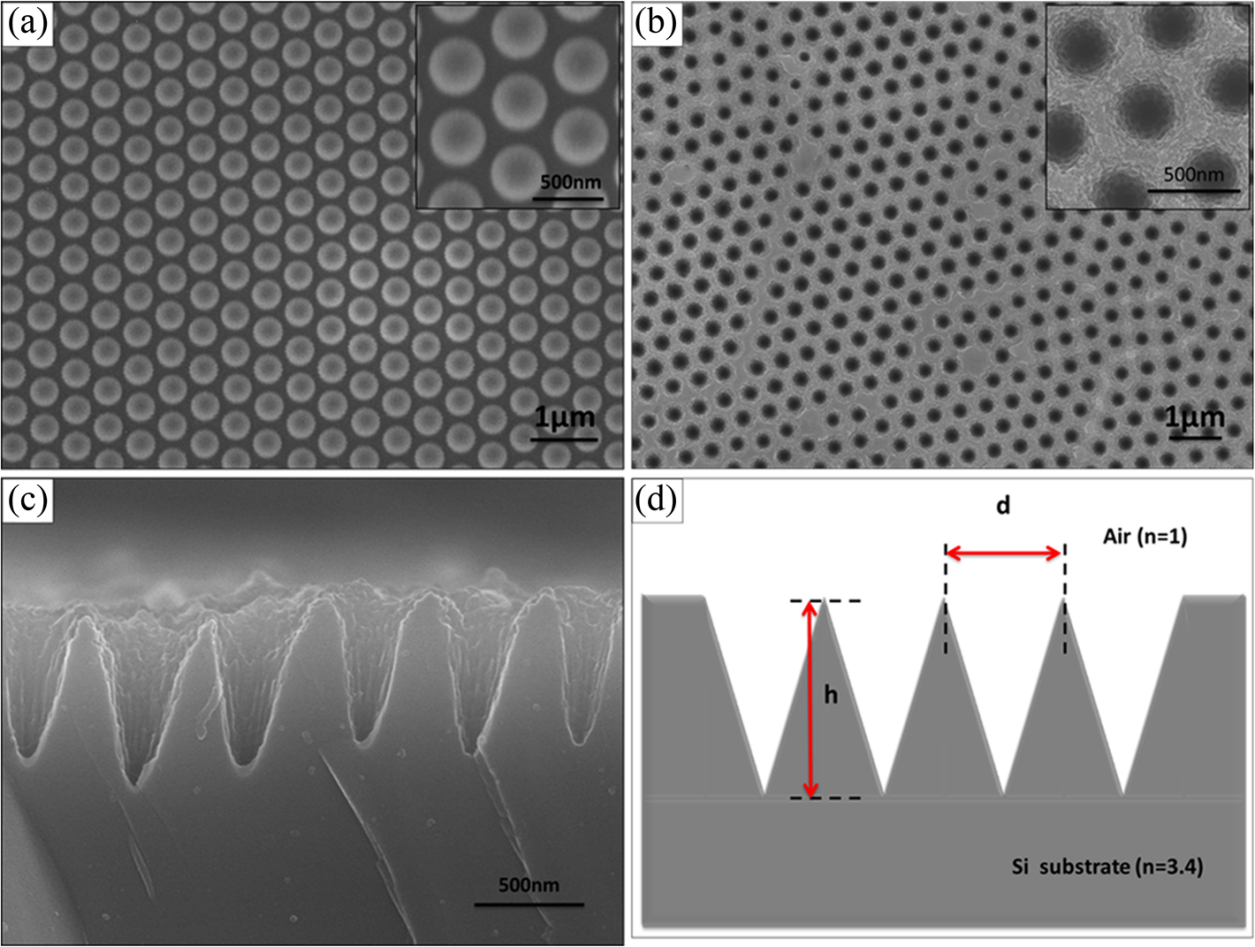
**SEM images and schematic drawing. (a)** SEM image of PS spheres on planar Si after etching with initial diameters of 500 nm. SEM images of ordered Si inverted nanocone arrays with spacing 500 nm, depth 800 nm **(b)** top-view, **(c)** cross-sectional view. **(d)** Schematic drawing of Si inverted nanocone arrays (depth *h*, diameter *d*).

Figure 2a shows the SEM image of the etched PS sphere pattern arrays. The initial diameter of the spheres was 500 nm. It can be seen that the PS spheres are periodically arranged on the surface in a large scale area. After 4 min of O_2_ plasma etching, the size of PS spheres was reduced to a specific diameter of about 390 nm. By using the Ni nanoholes as etching mask, highly-ordered Si nanohole arrays are obtained, as demonstrated in Figure 2b. The diameters and adjacent distances of the Si nanoholes are defined by the PS spheres. From the cross-sectional view in Figure 2c, the Si nanoholes show inverted cone shape. The depth of the holes is around 1 μm after 20 min of etching with 200 W of RIE power. The SEM images of PS spheres with diameters of 1,000 nm and Si inverted nanocone arrays with 1,000 nm pitches are provided in the Additional file (see Additional file 1: Figure S1 and S2). Figure 2d shows the schematic of the proposed inverted nanocone arrays. The lattice constant (period) of the hexagonal lattice is indicated as *a*. The diameter of the air hole at the top surface is *d* (*d* = *a*), and the depth is *h*.

**Figure 3:**
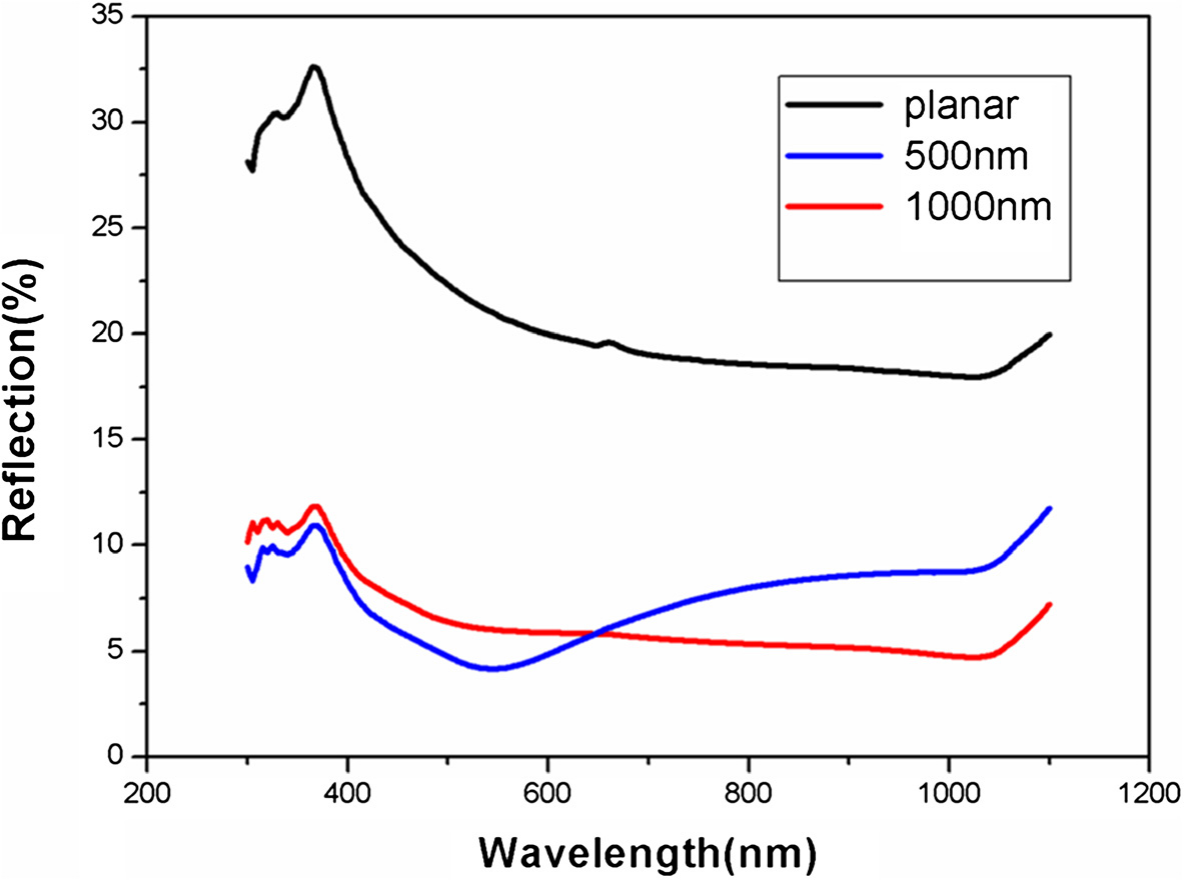
**Total hemispherical optical reflectance of planar Si and Si inverted nanocone arrays.** With different 500 and 1,000 nm pitches.

To investigate the anti-reflection properties of the Si inverted nanocone arrays, the diffuse reflectance spectrum were measured via a UV-vis spectrometer. For comparison, both the planar Si and ordered Si inverted nanocone arrays with different sizes were measured in a wavelength range of 300 to 1,100 nm. As shown in Figure 3, the Si inverted nanocone arrays can greatly suppress the reflection with wavelength above the Si bang gap (1.12 eV). The reflectance intensity of Si inverted nanocone arrays is less than 7% over broad range of 400 to 1,000 nm, much less than that of the planar Si (above 20%). For a closer observation of the reflectance spectrum of Si inverted nanocone arrays with 500 and 1,000 nm lattice in Figure 3, it can be seen that the Si inverted nanocone arrays yield different valleys of reflectance with the change of the sizes. Particularly, the Si inverted nanocone arrays with 500-nm lattice shows the lowest reflection around 542 nm wavelength, and the 1,000-nm lattice one demonstrates 4.7% reflection at approximately 1,020 nm wavelength, i.e., the Si inverted nanocone arrays can provide enhanced light trapping ability when the incident light wavelength is close to the sizes of the Si inverted nanocone.

The excellent broad antireflection properties of as-fabricated Si inverted nanocone arrays can be attributed the following two reasons. First, the gradient of effective index in the Si inverted nanocone arrays causes the incident light to be reflected at different depths from the interface of air and Si, as result of suppression of broad-band reflectance by destructive interferences [16]. The effective index (*n*_*eff*_) gradient of the Si inverted nanocones can be estimated by the following equation [17]: ${n}_{eff}={\left[f\;{n}_{S_i}^q+\left(1-f\right){n}_{air}^q\right]}^{1/\mathrm{q}}$ where *q* is 2/3, *n*_Si_ and *n*_air_ are the refractive indices of the Si and air, respectively, and *f* is the fill factor. Second, the periodical inverted nanocones might provide additional light trapping effect due to the optical diffraction.

**Figure 4:**
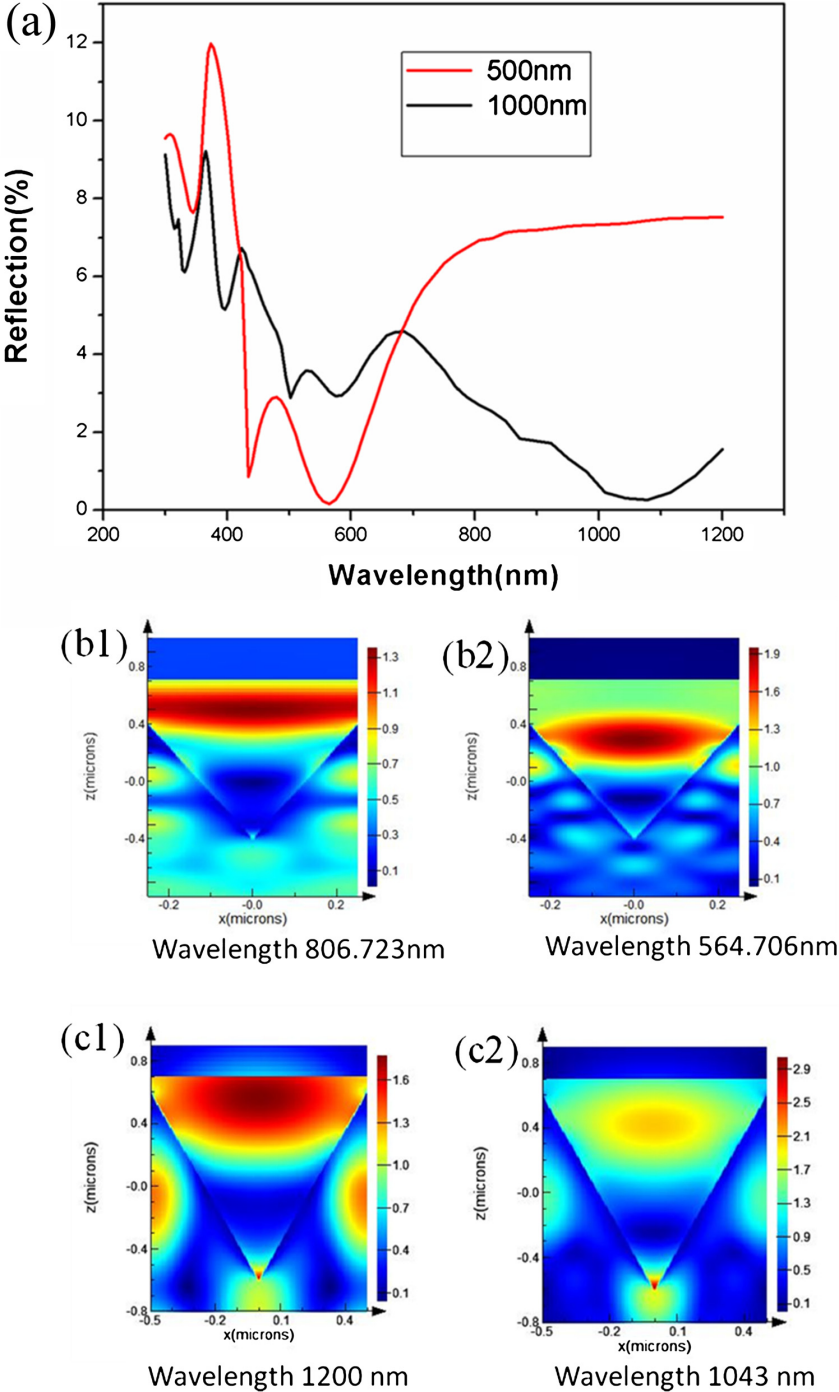
**Simulated reflectance spectrum and |E|** ^**2**^ **cross-sectional distribution of Si inverted nanocone arrays. (a)** The simulated reflectance spectrum of the Si inverted nanocone arrays with different 500 and 1,000 nm pitches. Simulated |E|^2^ cross-sectional distribution of Si inverted nanocone arrays: (b1 and b2) 500 pitch at 806.723 and 564.706 nm wavelength, respectively; (c1 and c2) 1,000 pitch at 1,200 and 1,043 nm wavelength, respectively.

In order to further verify the periodicity and sizes effects on the light trapping, FDTD simulations were performed on these Si inverted nanocone arrays with the hexagonal lattice model, resulting in the simulated reflection spectra as shown in Figure 4a. As expected, the simulated reflection spectra showed a quite consistent trend over all the wavelengths with the experimental ones as described in Figure 3. Obviously, the 500-nm periodicity Si inverted nanocone arrays show strong light capturing capability for approximately 500 nm wavelength light. Similar simulation result also occurred for the sample with 1,000-nm periodicity. Hence, the periodicity could lead to improved light capturing when the sizes match with the input wavelength. This phenomenon could be explained by the grating theory [18,19]. The diffraction of light in the periodic grating structure can increase the optical path length of photons, leading to increased absorption probability, which follows the grating equation [20]: *d*sin *θ* = *mλ*, where *d* is the grating lattice constant, that is the lattice constant of the Si inverted nanocone arrays, *θ* is the diffraction angle, *m* is the diffraction order, and *λ* is the incident light wavelength. When *d* is approaching *λ*, thus the *m*th order diffracted light will be propagating in plane inside the Si inverted nanocone array structures, as result of maximizing the light absorption probability.

In order to understand the electromagnetic (EM) wave coupling and propagation in the Si inverted nanocone array structures, the cross-sectional electric field intensity (|E|^2)^ distribution of the EM wave at different wavelengths was calculated in Figure 4b,c. In these four simulations, the EM plane waves propagate from top to bottom. The color index at the specific location reflects the magnitude of **|**E**|**^2^ at that point. For the 500-nm lattice Si inverted nanocone arrays (as shown in Figure 4 (b1 and b2)), the majority energy of EM wave at 564.706 nm are limited inside of the inverted nanocone arrays, while most of the EM wave at 806.723 nm are reflected. This observation can be attributed to the fact that the diameter of nanocone-hole is only 500 nm, which is much smaller than wavelength (*λ* = 806 nm). The **|**E**|**^2^ distributions of the 1,000-nm sample at 1,200 and 1,043 nm are shown in Figure 4c1 and c2, respectively. It can be found that most of the energy of EM waves around 1,000 nm are confined inside. The **|**E**|**^2^ distributions in the Si nanocone arrays with different pitches are well agreed with the optical diffraction theory, when the *d* is approaching *λ*, the highest order diffracted EM wave is propagating in plane inside the structures that can significantly improve the light coupling efficiency into Si inverted nanocone arrays.

## Conclusions

In summary, we have presented a simple and scalable method for the fabrication of highly-ordered inverted nanocone arrays with desirable diameters, depth, and pitches on crystalline silicon surface with colloid photolithography and RIE process. Compared to the planar Si, the inverted nanocone arrays structures exhibit outstanding broad anti-reflection properties in a wide spectrum range due to the gradient in the effective refractive index of nanocones and enhanced light trapping owing to optical diffraction. These inverted nanocone arrays might find application in nanostructured photovoltaic devices and photodetectors.

## Additional file

Additional file 1: Figure S1. Typical SEM images of PS spheres on planar Si after etching with initial diameters of 1,000 nm. **Figure S2.** SEM images of ordered Si inverted nanocone arrays with spacing of 1,000 nm (a) top-view, (b) cross-sectional view.
