# Supplementary material for: Highly-ordered silicon inverted nanocone arrays with broadband light antireflectance
Source: Nanoscale Res Lett. 2015 Jan 22;10:9. doi: 10.1186/s11671-014-0718-x (PMC4303704; doi:10.1186/s11671-014-0718-x)
Supplement: Additional file 1: Figure S1. — Typical SEM images of PS spheres on planar Si after etching with initial diameters of 1,000 nm. Figure S2. SEM images of ordered Si inverted nanocone arrays with spacing of 1,000 nm (a) top-view, (b) cross-sectional view. [file 11671_2014_718_MOESM1_ESM.docx]

**Supporting Information**

**Highly-ordered Silicon Inverted Nanocone Arrays with Broadband Light Antireflectance**

Dong Zhang,^1^ Weina Ren,^2^ Zhichao Zhu,^2^ Haifeng Zhang,^2^ Bo Liu,^2^ Wangzhou Shi ^1^, Xiaomei Qin,^1^* Chuanwei Cheng ^2^*

^1^Department of Physics, Shanghai Normal University, Shanghai, P. R. China. Email: xmqin@shnu.edu.cn

^2^Shanghai Key Laboratory of Special Artificial Microstructure Materials and Technology & School of Physics Science and Engineering, Tongji University, Shanghai 200092, P. R. China. Email: cwcheng@tongji.edu.cn


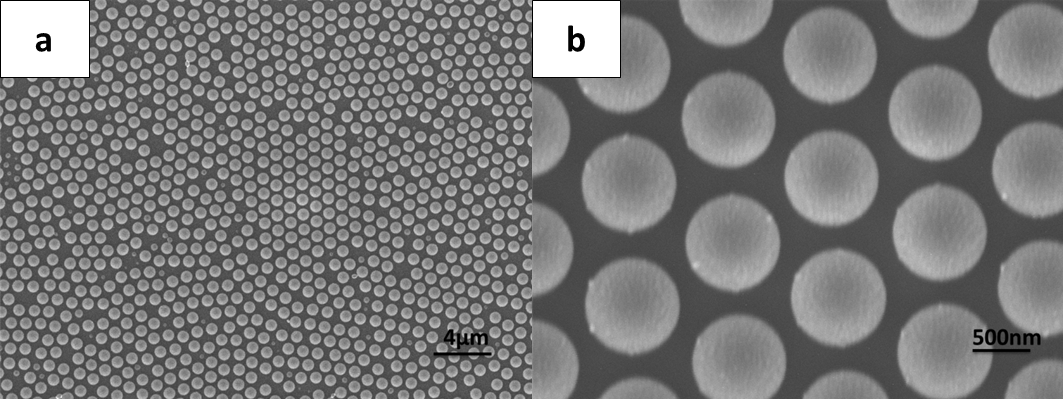


Figure S1 Typical SEM images of PS spheres on planar Si after etching with initial diameters of 1000 nm.


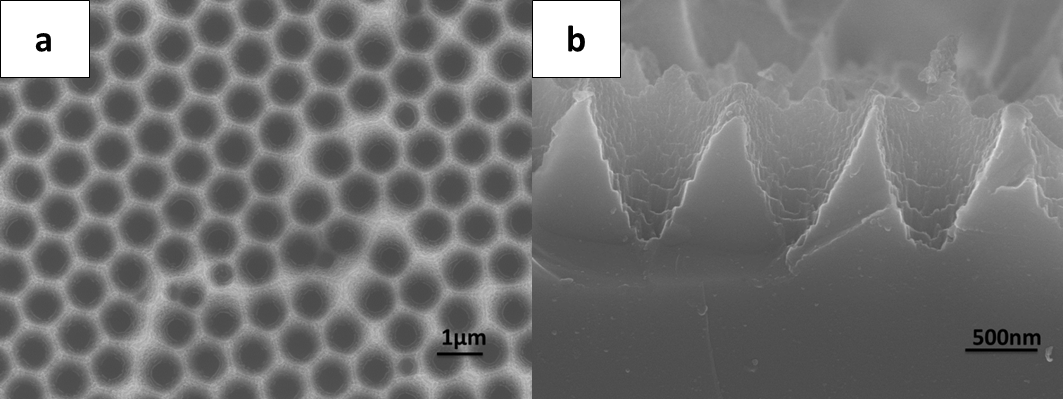


Figure S2 SEM images of ordered Si inverted nanocone arrays with spacing of 1000 nm (a) top-view, (b) cross-section view.
